# The Host Heat Shock Protein MRJ/DNAJB6 Modulates Virus Infection

**DOI:** 10.3389/fmicb.2019.02885

**Published:** 2019-12-11

**Authors:** Shih-Han Ko, Li-Min Huang, Woan-Yuh Tarn

**Affiliations:** ^1^Institute of Clinical Medicine, National Taiwan University College of Medicine, Taipei, Taiwan; ^2^Department of Pediatrics, National Taiwan University Children’s Hospital, National Taiwan University College of Medicine, Taipei, Taiwan; ^3^Institute of Biomedical Sciences, Academia Sinica, Taipei, Taiwan

**Keywords:** heat shock protein, Hsp40, MRJ, virus, morpholino oligonucleotide

## Abstract

A variety of pathogens take advantage of cellular heat shock proteins (HSPs) to complete their life cycle and exert pathogenic effects. MRJ (DNAJB6), a member of the heat shock protein 40 family, acts as a molecular chaperone for a wide range of cellular processes. MRJ mutations are linked to human diseases, such as muscular dystrophy and neurodegenerative diseases. There are two MRJ isoforms generated by alternative use of terminal exons, which differ in their C-terminus. This mini-review summarizes how these two MRJ isoforms participate differentially in viral production and virulence, and the possibility for MRJ as a therapeutic target.

## Introduction

Heat shock proteins (HSPs) function as molecular chaperones, thereby assisting protein folding, and non-covalent assembly or disassembly of macromolecules (Whitley et al., 1999). HSPs are structurally related proteins and classified based on their molecular weights, ranging from 10 to >100 kDa (Jee, 2016). HSP subfamily members exert similar functions across species. For example, small HSPs (HspB1 to HspB10) prevent the aggregation of misfolded proteins in an ATP-independent manner (Bakthisaran et al., 2015). ATP-dependent chaperones include Hsp60, Hsp70, and Hsp90. Hsp60 assists protein refolding throughout transport from the cytoplasm to the mitochondrial matrix (Cheng et al., 1989), while Hsp70 exerts the anti-aggregation activity with co-chaperones Hsp40 or Hsp110 (Kampinga and Craig, 2010). Hsp40 presents unfolded proteins to Hsp70 and stimulates its ATP hydrolysis (Bascos et al., 2017). Hsp90 regulates assembling, refolding and stabilizing of substrate proteins (Wandinger et al., 2008). HSPs function in a wide range of cellular processes to maintain protein homeostasis under physiological conditions and in response to environmental stresses (Hipp et al., 2019).

Invasion of pathogens, such as bacteria or viruses, may trigger cell stress responses and hence induces the production of cellular HSPs. Various viruses take advantage of cellular HSPs to overcome host environmental challenges and complete their infectious cycles (Neckers and Tatu, 2008). HSPs may participate in distinct steps during infection processes, such as viral entry, replication, and viral particle assembly and movement (Table 1). It is noteworthy that dengue virus particularly utilizes a set of Hsp70 family members for its entry, RNA replication and virion production (Taguwa et al., 2015). Moreover, some of the HSPs, particularly Hsp70, even become an integral component of virions (Santoro et al., 2009). All these findings emphasize the importance of HSPs in viral infection.

**TABLE 1:** Function of the HSP-virus interaction.

**HSP family**	**Member**	**Virus**	**Interacting viral proteins**	**Engrave roles in HSP-virus interaction**	**References**
Hsp40	DNAJA1, DNAJB1	IAV	NP, PB2, PA	Enhance nuclear import of vRNP complex and viral RNA synthesis	Cao et al., 2014; Batra et al., 2016
	DNAJA2	HCV	NS5A	NS5A-mediated IRES translation	Gonzalez et al., 2009
Hsp60	HSPD	HBV	HBx	Enhance HBx-induced apoptosis and HBx protein folding	Tanaka et al., 2004; Zhang et al., 2005
			Virus polymerase	Enhance viral polymerase activity	Park et al., 2002
Hsp70	HSPA1A	HIV	Virion	Virion assembly	Gurer et al., 2005
	HSPA1A	MuV	P protein	Facilitate IBs formation and modulate degradation of P protein	Katoh et al., 2015
	HSPA1A	HCV	NS5A	NS5A-mediated IRES translation	Gonzalez et al., 2009
	HSPA1, HSPA2, HSPA8	KSHV	RTCs	Facilitate RTCs formation and nuclear import	Baquero-Perez and Whitehouse, 2015
	HSPA5	RSV	Viral IBs	Enhance viral polymerase activity	Brown et al., 2005
	HSPA9	HBV	HBx	Viral protein folding	Zhang et al., 2005
Hsp90	HSP90AA1, HSP90AB1	IAV	PB1, PB2	Facilitate vRNP stabilization and nuclear import, viral RNA synthesis	Momose et al., 2002; Naito et al., 2007
	HSP90AA1	Rotavirus	NSP3	Viral protein folding and stabilization	Dutta et al., 2011
	HSP90A	RSV	Viral filaments and IBs	Viral particle assembly	Radhakrishnan et al., 2010
	HSP90	MuV	L protein	Viral protein stabilization	Katoh et al., 2017
	HSP90	HCV	NS3	Viral protein stabilization	Ujino et al., 2009

*IAV, influenza A virus; NP, nucleoprotein; vRNP, viral ribonucleoprotein complex; PB2, polymerase basic protein 2; PB1, polymerase basic protein 1; PA, polymerase acidic protein; HCV, hepatitis C virus; NS5A, non-structural 5A protein; HBV, hepatitis B virus; HBx, hepatitis B virus X protein; HIV, human immunodeficiency virus; MuV, mumps virus; P protein, phophoprotein; KSHV, kaposi’s sarcoma-associated herpes virus; RTCs, replication and transcription compartments; RSV, respiratory syncytial virus; IBs, inclusion bodies; NSP3, non-structural protein 3; L protein, large protein; NS3, non-structural protein 3.*

Heat shock proteins may also negatively impact viral infection. For example, two Hsp40 members inhibit the replication of human hepatitis B virus (HBV) (Sohn et al., 2006). Hsp70 interferes with nuclear import of the human immunodeficiency viruses (HIV) preinitiation complex, and viral gene expression and replication (Kumar et al., 2011). In addition, HSPs have immunomodulatory roles, although opposing. HSPs may act as a pro-inflammatory molecule by facilitating pathogenic antigen presentation on the antigen-presenting cells (Binder, 2014). On the other hand, HSPs may prevent immune activation by reducing inflammatory damages and promoting anti-inflammatory cytokines production (Hauet-Broere et al., 2006; Broere et al., 2011). Together, HSPs are engaged in both host immune response and viral pathogenesis during infection.

## The MRJ Protein and Its Functional Domains

**FIGURE 1:**
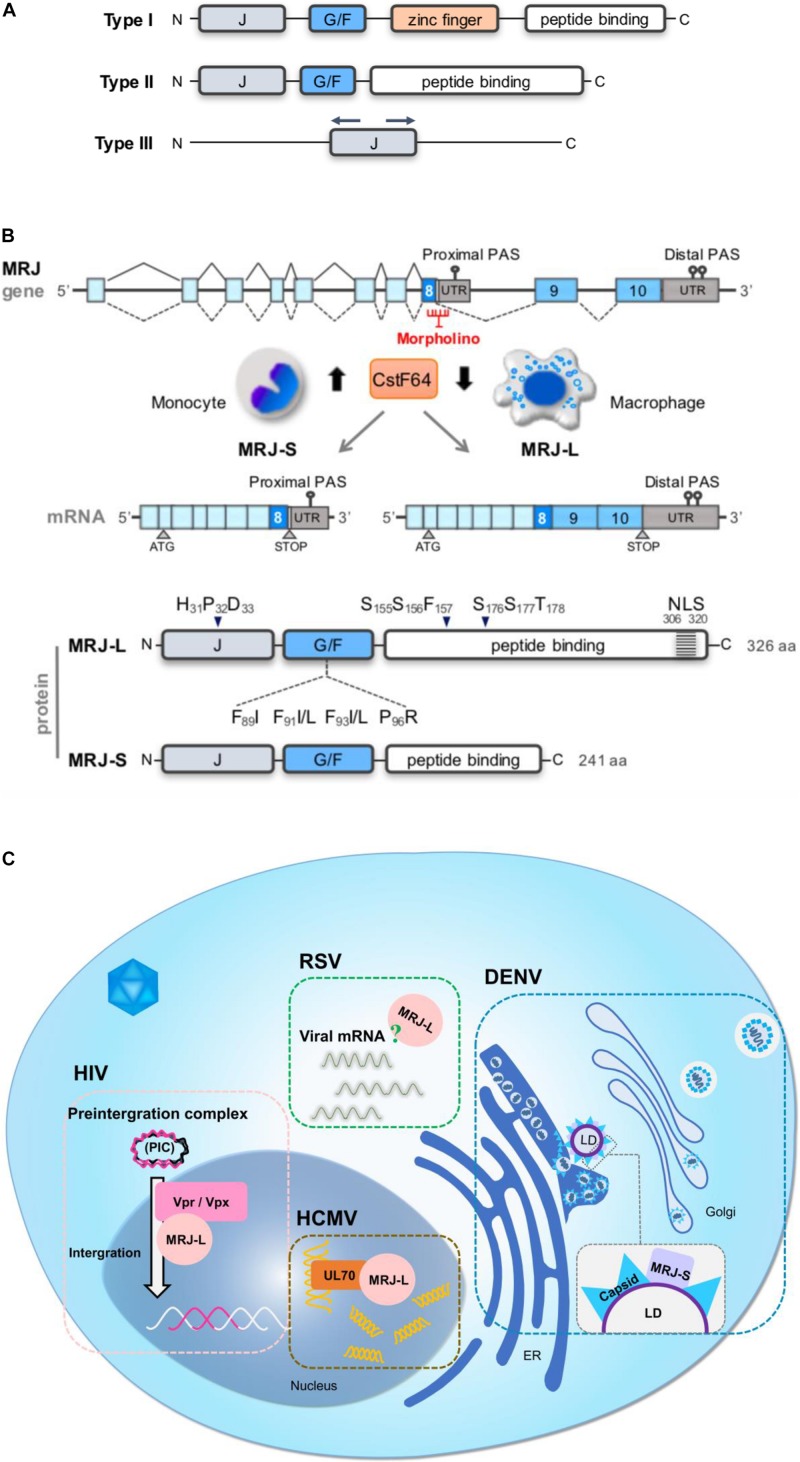
Human MRJ gene and functions in the viral life cycle. **(A)** Schematic diagram of three major types of Hsp40. All contain the J-domain. The J-domain is located at the N-terminus of type I and II Hsp40 but is found at various locations within the type III members. Additional domains are as depicted. **(B)** Schematic diagram (upper) shows genomic organization of the human MRJ gene and its transcript isoforms that are generated by alternative splicing and polyadenylation. A reduction in the CstF64 level in macrophages favors MRJ-L production. A morpholino oligonucleotide targeting the 5’ splice site of intron 8 suppresses (MRJ-L expression. The bottom schematic diagram shows three major domains of the MRJ protein, including the J-domain followed by the G/F-rich domain in the N-terminal part, and the C-terminal peptide-binding domain, which interacts with denatured polypeptides and also directs the function of Hsp70. The conserved HPD motif of the J-domain, the SSF and SST motifs (namely the S/T-rich region) in the C-terminal peptide-binding domain and LGMD-associated mutations in the G/F domain are indicated. MRJ-S has a truncated C-terminal domain that lacks the NLS. **(C)** The two MRJ isoforms participate in the infection of the following viruses. HIV, MRJ-L facilitates nuclear import of the PIC via its interaction with Vpr (HIV-1) or Vpx (HIV-2), and hence facilitates the integration of the HIV genome into host chromosomes. HCMV, MRJ-L interacts with the DNA polymerase UL70 of HCMV and facilitates its nuclear import so that MRJ-L enhances viral genome synthesis. RSV, MRJ-L is essential for the expression of viral mRNAs and proteins, and viral production, but how it functions remains yet unclear. DENV: DENV replicates its genomes released from the pore of DENV-induced vesicle-like structures. The newly synthesized genome is packaged in the nucleocapsid with the capsid protein, which subsequently buds form proximal ER membranes. MRJ-S is colocalized with the viral capsid protein on the LD surface and facilitates viral assembly. Assembled viruses are transported to the Golgi apparatus for the maturation processes.)

Mammalian relative of DnaJ (MRJ/DNAJB6) is an Hsp40 family member. The Hsp40 family can be categorized into three major types (I, II, and III), all of which share the ∼70-amino acid J-domain (Li et al., 2009; Figure 1A). In type-I Hsp40 proteins, the J-domain is at the N-terminus, followed by the glycine/phenylalanine (G/F)-rich region, the zinc finger domain, and the peptide-binding domain in the C-terminus. Type-II is similar to type-I but lacks a zinc finger domain. Type-III members contain only the J-domain, whose location differs between members (Qiu et al., 2006). Many, but not all, Hsp40 members act as cochaperones of Hsp70 by forming a heterodimer through the J-domain (Langer et al., 1992; Meacham et al., 1999; Lee et al., 2002). The J-domain of Hsp40 containing the invariant histidine-proline-aspartic acid (HPD) tripeptide stimulates the ATPase activity of Hsp70 and increases the affinity of Hsp70 for the polypeptide substrate released from Hsp40 (Summers et al., 2009). The G/F-rich region of MRJ contains several hydrophobic residues responsible for substrate recognition; phenylalanine mutations disrupt its anti-aggregation activity (Sarparanta et al., 2012; Palmio et al., 2015). The C-terminal part of MRJ contains a serine/threonine (S/T)-rich region, which is important for substrate binding (Kakkar et al., 2016). Nevertheless, Hsp40s bind and transfer non-native polypeptides to Hsp70 through distinct mechanisms, which are subject to further processing (Summers et al., 2009). In addition, the C-terminal region of MRJ is also involved in polydisperse oligomeric complexes and oligomerization (Hageman et al., 2010; Figure 1B, protein). All these functional domains are present in both splice isoforms of MRJ (see below).

## Regulation of MRJ Isoform Expression

MRJ is ubiquitously expressed in human tissues, with a higher level in the brain (Chuang et al., 2002). MRJ is upregulated during mitosis in HeLa cells perhaps to support mitotic activities (Dey et al., 2009). Human *MRJ* has two splice isoforms, *MRJ-L* and *MRJ-S*, generated through alternative splicing (Hanai and Mashima, 2003). The *MRJ* gene has ten exons. The first eight exons are included in both isoforms, while the last two exons are missing in *MRJ-S* (Ko et al., 2018; Figure 1B, mRNA). MRJ-L and MRJ-S proteins are comprised of 326 and 241 amino acid residues, respectively; both possess the aforementioned three functional domains. MRJ-L has a nuclear localization signal (NLS) in its very C-terminal region. Suppression of splicing in conjunction with activation of aberrant polyadenylation signals in intron 8 leads to *MRJ-S* expression. *MRJ* has been identified as a potential target transcript of cleavage stimulation factor subunit 2 (CstF64/CSTF2), a cleavage stimulation factor for mRNA 3’-end processing (Yao et al., 2012). Knockdown or overexpression of CstF64, respectively, increases and decreases the L/S isoform ratio (Ko et al., 2018). A decline in the CstF64 level during macrophage differentiation favors *MRJ-L* expression (Figure 1B, mRNA). In addition, serine/arginine-rich splicing factor 3 (SRSF3) may promote *MRJ-S* expression (Ko et al., 2018). Cyclin-dependent kinase 12 (CDK12) amplification in breast cancer results in downregulation of *MRJ-L* via modulating its terminal exon selection (Tien et al., 2017). Thus, the *MRJ* isoform ratio may be modulated in response to different cellular signals. Moreover, *MRJ-L* expression can be enhanced by increasing the strength of the 5’ splice site of intron 8. Single nucleotide variations in the proximal polyadenylation signal and the polypyrimidine tract of intron 8 also affect *MRJ* isoform ratios. Thus, both alternative splicing and alternative polyadenylation activities determine *MRJ* isoform expression (Ko et al., 2018).

## Cellular Functions of MRJ

*MRJ* knockout mice show embryonic lethality due in part to placental abnormalities and neural tube defects (Hunter et al., 1999; Watson et al., 2009). MRJ is involved in a variety of physiological processes, from transcription, cellular signaling to cell adhesion. MRJ suppresses the transcriptional activity of nuclear factors of activated T-cells (NFAT) by recruiting class II histone deacetylases, and hence, reduces calcineurin-induced cardiac myocyte growth. This observation suggests a role of MRJ in preventing cardiac hypertrophy (Dai et al., 2005). More notably, MRJ suppresses Wnt/β-catenin signaling through multiple pathways. Essentially, MRJ upregulates the secretary glycoprotein and Wnt inhibitor dickkopf 1 (DKK1) and maintains the dephosphorylation status of glycogen synthase kinase 3β (GSK3β) through the protein phosphatase PP2A and hence promotes degradation of β-catenin (Meng et al., 2016). This suppressive effect of MRJ on Wnt-β-catenin signaling negatively regulates tumor growth and metastases. Accordingly, a reduction of the MRJ level is present in various invasive and metastatic cancers as earlier mentioned. On the other hand, MRJ influences cytoskeletal organization, which is responsible for cell growth, division, and migration. For example, MRJ modulates intermediate filament organization via its direct interaction with keratins (Izawa et al., 2000). *MRJ* knockout causes actin cytoskeletal collapse in chorionic trophoblast cells (Watson et al., 2011). MRJ also contributes to cell adhesion and migration via its interaction with urokinase-type plasminogen activator receptor (uPAR) (De Bock et al., 2010; Lin et al., 2014). A recent report reveals that MRJ promotes spindle pole focusing via its interaction with dynactin, which is essential for chromosome segregation during cell division (Rosas-Salvans et al., 2019).

## Pathological Effects of Defective MRJ

Genetic mutations or dysfunction of MRJ have been observed in human diseases such as limb-girdle muscular dystrophy (LGMD), myopathy and neurodegenerative diseases. Phenylalanine mutations in the (G/F)-rich region of MRJ are found in LGMD and distal myopathy, indicating that the chaperone activity of MRJ is critical for preventing proteinopathy (Harms et al., 2012; Sarparanta et al., 2012; Li et al., 2016; Jonson et al., 2018). MRJ mutations result in myofibrillar aggregates containing ubiquitin, ubiquitin-binding protein p62 and TAR DNA-binding protein 43 (TDP-43) (Sato et al., 2013; Sandell et al., 2016). Notably, TDP-43 aggregation is a characteristics of amyotrophic lateral sclerosis (Tamaki et al., 2018), emphasizing the pathological effect of defective MRJ in neurodegenerative disorders. The C-terminal S/T-rich region in MRJ exhibits the suppressive effect on the formation of different aggregation-prone peptides such as amyloid-β and polyglutamine peptides that are involved in the pathogenesis of Alzheimer’s disease and Huntington’s disease, respectively (Kakkar et al., 2016; Mansson et al., 2018; Bason et al., 2019). MRJ has also been implicated in Parkinson’s disease. Upregulation of MRJ in parkinsonian astrocytes prevents the neuronal release of α-synuclein/SNCA, which has the potential to form toxic aggregates, suggesting a protective role of MRJ (Durrenberger et al., 2009; Aprile et al., 2017). A more recent study indicates that the chaperone activity of MRJ also suppresses mutant parkin aggregation (Kakkar et al., 2016). Thus, it is likely that the chaperone function of MRJ contributes to preventing protein misfolding in neurodegenerative diseases.

## Role of MRJ in Virus Infection

In addition to the cellular functions above mentioned, both MRJ isoforms have been implicated in infection and pathogenesis of multiple human viruses. A recent report unveils the involvement of a translocon complex factor, Sec61, in the biogenesis of several different viral proteins, suggesting that targeting Sec61 can provide an antiviral strategy against multiple viruses (Heaton et al., 2016). In light of this finding, we review the roles of MRJ in infection and propagation of several viruses and discuss the potential of targeting MRJ as an antiviral strategy.

### Human Immunodeficiency Viruses (HIV)

Human immunodeficiency viruses is a retrovirus that causes acquired immunodeficiency syndrome (AIDS), which destroys the immune system of infections (Sharp and Hahn, 2011). HIV infects macrophages and CD4^+^ T helper cells through the CD4 receptor and its coreceptor, i.e., chemokine receptor CCR5 or CXCR4 (Maartens et al., 2014). After infection, HIV is integrated into the human genome, which is essential for the viral life cycle (Moir et al., 2011). For integration, HIV establishes the pre-integration complex (PIC), consisting of the cDNA that is converted from its genomic RNA and several cellular and viral proteins including the viral protein R (Vpr). Vpr participates in proviral integration into the host genome (Chiang et al., 2014; Pirrone et al., 2014). MRJ-L facilitates nuclear localization of the HIV-1 pre-integration complex via its direct interaction with Vpr (Chiang et al., 2014). As compared to MRJ-L, MRJ-S displays a relatively weak activity in nuclear localization of Vpr/Vpx likely due to its lack of the C-terminal NLS of MRJ-L (Cheng et al., 2008; Chiang et al., 2014). Notably, mutations in the HPD motif of MRJ-L disrupt the activity of MRJ-L in facilitating Vpx (or HIV) nuclear import, indicating the involvement of Hsp70 (Cheng et al., 2008). Analogously, MRJ-L assists nuclear import of the HIV-2 viral protein X (Vpx), the paralog of HIV-1 Vpr. Depletion of MRJ-L restricts HIV-2 replication due to reduced nuclear import of the PIC (Cheng et al., 2008). On the other hand, overexpression of MRJ-S suppresses HIV proviral transcription and hence compromises HIV-1 production (Urano et al., 2013). These observations together suggest that a higher L/S ratio of MRJ may promote HIV infection (Figure 1C, HIV). A cohort study reveals that HIV-infected individuals indeed exhibit a slightly higher level of MRJ-L in macrophages than healthy subjects (Chiang et al., 2014), supporting the positive role of MRJ-L in HIV-1 infection. It is speculated that *cis*-element polymorphisms of *MRJ* that favor L isoform expression may increase the probability of HIV infection (Ko et al., 2018). Therefore, the MRJ-L level difference between individuals may predict HIV susceptibility. On the other hand, the negative regulatory factor (Nef) of HIV facilitates nuclear translocation of Hsp40, which subsequently facilitates viral gene expression. Nevertheless, Hsp70 can counteract the nuclear import of Vpr-mediated PIC complex and hence inhibits viral replication (Iordanskiy et al., 2004).

### Human Cytomegalovirus (HCMV)

Human cytomegalovirus is a common opportunistic pathogen that may establish long-life latency without any symptoms in healthy individuals but may threaten immunocompromised individuals and neonates (Kenneson and Cannon, 2007). HCMV has the largest genome among the human herpesviruses and replicates in the nucleus of cells. The HCMV DNA-dependent RNA polymerase, i.e., the primase UL70, forms the helicase-primase complex with UL102/105 to synthesize short RNA primers for viral DNA replication (McMahon and Anders, 2002). MRJ-L interacts with UL70 through a fragment containing the G/F-rich region and facilitates nuclear entry of UL70, thereby promoting viral DNA synthesis (Figure 1C, HCMV). On the other hand, MRJ-S is co-localized with the primase in the cytoplasm that reduces viral genome expression and synthesis (Pei et al., 2012). Thus, MRJ isoforms differentially modulate HCMV replication. Reduction of the MRJ-L expression level conceivably inhibits viral lytic infection and can be used as an anti-HCMV strategy (Biron, 2006).

### Respiratory Syncytial Virus (RSV)

Human RSV causes lower respiratory tract infection in infants and children worldwide. RSV infection shows a higher risk of mortality compared to seasonal influenza infection in elder individuals (Kwon et al., 2017). RSV belongs to the Paramyxoviridae family, consisting of a negative-sense single-stranded RNA genome that replicates in the host cytoplasm. The viral RNA-dependent RNA polymerase is responsible for both viral transcription and replication (Noton et al., 2019). Intriguingly, knockdown of MRJ-L reduces viral mRNA and protein expression and virion production, while depletion of MRJ-S has no such effects (Ko et al., 2018), indicating the critical role of MRJ-L in RSV propagation. Nevertheless, whether MRJ-L interferes with the RNA polymerase activity of RSV remains to be determined. Additionally, whether the nuclear localization property of MRJ-L is required for RSV viral production also remains puzzling (Figure 1C, RSV). If this were the case, it would be interesting to elucidate why an RNA virus, which completes its life cycle in the cytoplasm, requires the nuclear function(s) of MRJ-L for propagation.

### Dengue Virus (DENV)

Dengue virus is a mosquito-transmitted pathogen and its infection may cause haemorrhagic fever (Brady et al., 2012; Bhatt et al., 2013). DENV is a member of the Flaviviridae family with a positive-sense single-stranded RNA genome. Viral genome replication and package solely occur in the host cytoplasm. DENV infection induces autophagy that targets cellular lipid droplets (LDs), which are endoplasmic reticulum-derived storage organelles of neutral lipids, and hence stimulates lipid metabolism (Randall, 2018). On the other hand, LDs are essential for DENV production. During virion assembly, the DENV capsid protein binds to an LD surface protein and forms the nucleocapsids with viral genomes in the endoplasmic reticulum (Samsa et al., 2009; Heaton and Randall, 2010). HSPs participate in multiple steps in the DENV life cycle (Taguwa et al., 2015). Among them, MRJ-S is colocalized with the capsid protein on LDs and aids viral particle assembly (Taguwa et al., 2015; Figure 1C, DENV). Depletion of MRJ-S impairs viral RNA replication and virion production (Taguwa et al., 2015). MRJ-S with mutations in the HPD motif fails to rescue viral production in MRJ-S-depleted cells, indicating the cooperative role of MRJ-S and Hsp70 in viral particle biogenesis. Nevertheless, MRJ-L is not engaged in the process of DENV propagation (Taguwa et al., 2015).

## Targeting MRJ as an Antiviral Strategy

Antisense morpholino oligonucleotides targeting viral RNAs or host mRNAs that encode proteins essential for viral propagation have been designed for treatment of viral infection (Warren et al., 2012). For example, a splice switching morpholino oligonucleotide can restrict influenza viral replication by suppressing exon inclusion of the host *transmembrane serine protease 2 (TMPRSS2)* (Bottcher-Friebertshauser et al., 2011). In light of the findings that MRJ is involved in viral propagation, it is possible to interfere with viral infection by targeting MRJ or modulating its splice isoform expression. Depletion of *MRJ-L* by siRNAs inhibits viral life cycles of HCMV and HIV (Cheng et al., 2008; Pei et al., 2012). Our recent report shows that a morpholino oligonucleotide complementary to the 5’ splice site of *MRJ* intron 8 efficiently inhibits *MRJ-L* expression *in vitro* (Ko et al., 2018). This morpholino disrupts the propagation of both pesudotyped and native HIV-1 in macrophage-like cells, and also effectively restricts subgenomic synthesis of RSV (Ko et al., 2018). It is likewise possible that masking the polyadenylation signal in intron 8 can suppress *MRJ-S* production. Since flaviviruses share a similar viral processing mechanism in LDs, it would be interesting to know whether MRJ-S-targeting agents may have a broad-spectrum antiviral effect. Since small molecule splicing modulators developed recently demonstrate their therapeutic potentials (Bates et al., 2017), it is worthy to evaluate whether any of them could influence MRJ isoform ratios, and hence impact viral infection. As described above, MRJ acts as an efficient suppressor of polyglutamine aggregation (Chuang et al., 2002). Therefore, harnessing the expression or chaperone activity of MRJ would be used for treatment of neurodegenerative disorders. Together, MRJ holds a great potential as drug targets.

## Conclusion

It has been demonstrated that single nucleotide polymorphisms near splice sites or polyadenylation sites of MRJ affect its isoform expression ratio (Ko et al., 2018). Thus, individuals may vary their susceptibility to viral infection, cancer or other disorders. Although the chaperone activity of MRJ is likely important for protein proteostasis, particularly disease-causing proteins, it is yet unclear whether this activity is critical for its various functions in viral infection. Nevertheless, the importance of the MRJ HPD motif in supporting HIV and dengue viral production suggests that the ATPase activity of Hsp70 contributes to viral propagation. Identification of small molecule compounds that selectively target HSPs has the values in prevention and treatment of viral infection. It has been demonstrated that Hsp70 inhibitors exert substantial antiviral activities against DENV as well as other flaviviruses (Taguwa et al., 2015, 2019). Small molecules targeting different domains of Hsp90 or interfering with its cochaperone or substrate protein binding have also shown the potential in therapeutic treatment of cancer or neurodegenerative disorders (Shrestha et al., 2016). However, pharmacologically manipulating the activity of Hsp40 is not yet available. Therefore, to have a better understanding of the isoform expression and domain structure-function relations of MRJ would be important for drug design toward viral infection.

## Author Contributions

S-HK and W-YT designed the figures and wrote the manuscript. L-MH and W-YT edited the manuscript.

## Conflict of Interest

The authors declare that the research was conducted in the absence of any commercial or financial relationships that could be construed as a potential conflict of interest.
